# Genomic diversity, inbreeding, and selection signatures in duroc, landrace, and yorkshire pigs from a long-term closed breeding system

**DOI:** 10.1007/s13353-026-01072-9

**Published:** 2026-06-03

**Authors:** Huangyi Tang, Henrique A. Mulim, Shi-Yi Chen, Allan P. Schinckel, Hongfeng-Xue, Dingjie-Qiu, Yizhan-Liu, Hinayah Rojas de Oliveira

**Affiliations:** 1 Purdue University, West Lafayette, IN 47906 USA; 2 Farm Animal Germplasm Resources and Biotech Breeding Key Laboratory of Sichuan Province, Sichuan Agricultural University, Chengdu, Sichuan 611130 China; 3 Fujian Yongcheng Breeding Technology Group Co., Ltd, Fuqing, 350319 Fujian China

**Keywords:** Generation proxy, Genetic diversity, Inbreeding, Linkage disequilibrium, Principal component, Runs of homozygosity

## Abstract

Duroc (DD), Landrace (LL), and Yorkshire (YY) are among the most widely used commercial pig breeds, having undergone intense long-term selection within closed breeding systems. This study presents a comprehensive genomic analysis of genetic diversity, inbreeding patterns, and selection signatures in DD, LL, and YY populations that have been subject to close breeding for over 15 years. Genomic and pedigree data were available for 1,088 animals (DD = 348, LL = 276, YY = 464), genotyped using the GenoBaits^®^ Porcine 100 K SNP panel. Principal component analysis and genetic diversity metrics revealed distinct population structures among the three breeds. Pairwise genetic differentiation supported this pattern, with DD showing the greatest divergence from LL (0.34 ± 0.24) and YY (0.33 ± 0.24), while LL and YY were more closely related (FST = 0.22 ± 0.19). Linkage disequilibrium (LD) analysis further confirmed these differences, as DD exhibited the highest average r² (0.34), followed by LL (0.28) and YY (0.25). Within-breed genetic diversity metrics, including observed heterozygosity (HO: 0.37 in DD, 0.39 in LL, 0.38 in YY), expected heterozygosity (HE: 0.36 in DD, 0.37 in LL, 0.38 in YY), and minor allele frequency (MAF: 0.27 in DD, 0.28 in LL, 0.29 in YY), indicated greater genetic variability in LL and YY compared to DD. Runs of homozygosity (ROH) analyses revealed different patterns of autozygosity, with DD exhibiting more long ROH indicative of recent inbreeding, while YY harbored a higher number of short ROH, suggestive of more ancient demographic events. ROH-based inbreeding coefficients (FROH) consistently exceeded pedigree-based estimates (FPED) across all breeds, highlighting the presence of recent or unrecorded inbreeding that pedigree data may not fully capture. According to Generation Proxy Selection Mapping (GPSM), 17, 1, and 12 significant SNPs were detected in DD, LL, and YY, respectively. Functional annotation of ROH islands and GPSM-significant loci revealed both breed-specific and overlapping QTLs related to traits such as growth, reproduction, and carcass. In general, the findings of this study contribute to a deeper understanding of the genomic consequences of long-term closed breeding and provide reference information to support consideration of breeding strategies that balance continued selection for productivity with the maintenance of genetic diversity in modern commercial pig populations.

**Supplementary Information:**

The online version contains supplementary material available at https://doi.org/10.1007/s13353-026-01072-9.

## Introduction

Pork represents the most widely consumed meat, accounting for approximately 36% of worldwide meat production and serving as a critical source of protein in many countries (Mateos et al. 2024). The genetic management of highly performing commercial pig breeds directly influences both productivity and long-term sustainability of pork production systems. Among commercial pig breeds, Duroc (DD), Landrace (LL), and Yorkshire (YY) have achieved widespread adoption due to their complementary roles (Choi et al. 2014): DD serves as a premier terminal sire breed, enhancing growth and carcass traits (Badke et al. 2012). In contrast, LL and YY serve as maternal lines with superior reproductive performance and exceptional maternal characteristics (Aguilar and Misztal 2008; Xie et al. 2023).

However, intensive selection practices that have driven remarkable productivity gains in commercial pig breeding have simultaneously raised significant concerns about genetic diversity loss (Hulsegge et al. 2019). Commercial breeding programs typically operate as closed or semi-closed systems with limited genetic material exchange to maintain biosecurity and preserve economically valuable genetic combinations. While this approach ensures consistent performance and reduces disease risks, it also creates conditions that may accelerate the erosion of genetic diversity through genetic drift, inbreeding, and intense directional selection for production traits (Zorc et al. 2022). Reduced genetic diversity potentially compromises long-term population adaptability to emerging diseases, environmental changes, and evolving consumer preferences (De Roo 1988).

Advanced genomic tools now enable comprehensive analysis of genetic diversity through multiple complementary approaches, providing deeper insights into underlying population genetic dynamics. For instance, runs of homozygosity (ROH) are continuous stretches of homozygous genotypes inherited from common ancestors, serving as valuable indicators for assessing inbreeding levels and reconstructing demographic history (Peripolli et al. 2018). The length of ROH provides insight into the timing of inbreeding events, with shorter ROH indicating more ancient events and longer ROH reflecting recent consanguineous mating (Kirin et al. 2010). ROH distribution patterns are significantly influenced by selection pressures, creating “ROH islands”. ROH islands are defined as an intense concentration of ROH in specific genomic regions, i.e., regions experiencing strong selective pressure (Yang et al. 2012).

While ROH patterns effectively identify genomic regions shaped by historical selection events, they provide limited information about the temporal dynamics of genetic changes (Liu et al. 2024). To overcome this limitation, a novel method called birthdate selection mapping (BDSM) was first introduced in 2012, which uses birth year or pedigree-based generation numbers as response variables to elucidate temporal trends in allele frequency shifts (Decker et al. 2012). The method was later refined and formally renamed generation proxy selection mapping (GPSM), which enables detection of loci under directional selection over time (Rowan et al. 2021). Despite the independent application of GPSM and ROH approaches in livestock populations, few studies have integrated these approaches to jointly investigate recent selection patterns and long-term genomic homozygosity, particularly in core commercial pig breeds (Grohmann et al. 2023). Furthermore, no studies have examined these patterns in contemporary animals from commercial breeding systems operating under closed conditions for extended periods, where the cumulative effects of restricted gene flow and intensive selection may be most pronounced. This study addresses this knowledge gap by leveraging genomic and pedigree data from contemporary DD, LL, and YY pigs from a commercial breeding company operating as a closed herd for over 15 years. The specific objectives of this study are to: (1) characterize population structure and genetic diversity to quantify current genetic variation and demographic parameters within each breed; (2) assess inbreeding levels and ROH patterns in each breed; (3) identify genomic regions under selection and reconstruct the recent selection trends within each breed by applying the GPSM approach; and (4) interpret the functional consequences of long-term closed breeding by analyzing genes and pathways in regions jointly identified by ROH islands and GPSM. The findings from this study will contribute to understanding genetic diversity patterns in contemporary commercial pig populations following extended periods of closed breeding.

## Materials and methods

This study was conducted using pre-existing datasets provided by commercial breeding facilities. Therefore, as no animal procedures were performed for this research, Institutional Animal Care and Use Committee (IACUC) approval was not required.

### Dataset description, source, and quality control

This study used genomic and pedigree datasets that were provided by Fujian Yongcheng Breeding Technology Group Corporation (Fuqing, Fujian Province, China) and originated from two breeding farms: Qingliu Yongcheng (FQYC) and Guizhou Yongcheng (GZYC). The dataset included animals born between 2009 and 2024, representing multiple generations from long-term closed breeding populations. A total of 1,088 pigs were genotyped (348 Duroc, 276 Landrace, and 464 Yorkshire), including 303 males and 785 females. Genotyping was initiated in 2023 and focused on a random sample of animals born between 2020 and 2024. The corresponding pedigree data encompassed 114,050 DD, 181,104 LL, and 326,289 YY animals, with complete sire and dam information available for 99.8%, 99.5%, and 99.7% of individuals, respectively. The average number of discrete generation equivalents of 7.96 for DD, 7.36 for LL, and 6.88 for YY. The animals were genotyped by using GenoBaits^®^ Porcine 100 K Panel (MOLBREDING, ShijiaZhuang, China). Quality control was performed using PLINK v1.9 (Purcell et al. 2007), and specific filtering criteria were applied for different analyses, as detailed in Table 1.

**Table 1:** Summary of genotypic quality control (QC) by breed

QC Steps	DD	LL	YY	Analyses
Original Markers Without Sex Chromosome	7,9901			
Removing SNPs with call rate < 0.95	2,129	2,807	2,580	PCA, ROH, LD, NE, GPSM
Removing SNPs with HWE < 10^− 6^	148	176	226	LD, NE
Removing individuals with call rate < 0.9	0	0	0	PCA, ROH, LD, NE, GPSM
Removing SNPs with MAF < 0.05	28,267	18,317	14,136	LD

Breeds are Duroc (DD), Landrace (LL), and Yorkshire (YY). Analyses are: Principal component analysis (PCA), Runs of homozygosity (ROH), Linkage disequilibrium (LD), Effective population size (NE), Generation proxy selection mapping (GPSM).

### Linkage disequilibrium, consistency of gametic phase, and effective population size

Linkage disequilibrium (LD, r²) was estimated for each breed using PLINK v1.9 (Purcell et al. 2007), and it defined as the squared correlation between alleles at two loci following Hill and Robertson (Hill and Robertson 1968). The LD decay was evaluated using PopLDdecay (Zhang et al. 2019). Effective population size (Ne) was estimated using SNeP (Barbato et al. 2015), based on the method described by(Corbin et al. 2012). To evaluate whether the breeds have the potential to be combined for genomic selection, the consistency of the gametic phase (CGP) between breeds was assessed. Consistency of the gametic phase (CGP) between breeds was assessed by calculating the Pearson correlation coefficients between signed square roots of r² values, computed within 10-kb distance bins ranging from 0 to 1,000 kb.

### PCA and genetic diversity metric

To assess the genetic relatedness among populations, Principal Component Analysis (PCA) was performed using the shared SNPs across the three breeds with PLINK v1.90 (Purcell et al. 2007). The principal components were derived from the standardized variance of the genomic relationship matrix (**G**), where the covariance of each SNP was divided by its respective variance. The **G** was created according to the first method published by VanRanden (VanRaden 2008). The PCA results generated by PLINK v1.9 (Purcell et al. 2007) were subsequently visualized using the R software (Team 2024). Observed heterozygosity (HO) and expected heterozygosity (HE) were also calculated using PLINK v1.9 (Purcell et al. 2007). The average pairwise genetic distance (DST) separating individuals within each population was also calculated using PLINK v1.9 (Purcell et al. 2007). Additionally, the average MAF was calculated to assess allelic diversity within each population. Pairwise estimates of genetic differentiation based on fixation index (FST) between populations were computed using PLINK v1.9 (Purcell et al. 2007), based on the method of Weir and Cockerham (Weir and Cockerham 1984).

### ROH detection and classification

Runs of Homozygosity (ROH) were inferred using the PLINK v1.9 software (Purcell et al. 2007), with detection parameters aligned with those described by (Wen et al. 2025). The detection criteria included: (1) a minimum length of 1 Mb; (2) inclusion of at least 50 consecutive SNPs within each segment; (3) a maximum inter-marker gap of 1 Mb between consecutive SNPs; (4) a minimum SNP density of one marker per 100 kb; (5) a sliding window of 50 SNPs moving forward by one SNP at each step across the genome; and (6) allowance of up to five missing genotypes and a maximum of one heterozygous genotype per ROH. All identified ROH segments were classified into five length categories: <2 Mb, 2–4 Mb, 4–8 Mb, 8–16 Mb, and > 16 Mb. The ROH islands were identified by calculating the proportion of individuals in which each SNP was located within a ROH, and genomic regions where SNPs occurred within ROHs in more than 80% of the population were defined as ROH islands (Peripolli et al. 2018). This threshold was selected following a similar approach reported in a previous study (Gorssen et al. 2021).

### Inbreeding coefficient estimation

A total of seven approaches were applied to estimate inbreeding coefficients in this study. The first approach was pedigree-based inbreeding (FPED), which was calculated using the inbupgf90 program from the BLUPF90 software family (Aguilar and Misztal 2008). Pedigree inbreeding coefficients were computed following the approach proposed by Meuwissen and Luo (Meuwissen and Luo 1992), using the tabular method originally proposed by Lush (Lush 2010). The second approach was genomic relationship matrix–based inbreeding (FGRM), calculated according to the method proposed by (VanRaden 2008). The third and fourth approaches were homozygosity-based inbreeding coefficients, FHOM and FHET, as described by (Purcell et al. 2007). The fifth approach, FHOM2, is a SNP-level homozygosity-based inbreeding coefficient proposed by (Yang et al. 2011). The sixth approach, FUNI, estimates inbreeding based on the correlation between uniting gametes, following the model proposed by (Yang et al. 2010). Finally, runs of homozygosity–based inbreeding (FROH) was calculated as the total length ROH divided by the total length of the autosomal genome, following the approach described by(McQuillan et al. 2008). The seven methods of inbreeding coefficients were eventually compared within DD, LL, and YY using Pearson correlation coefficients.

### Generation proxy selection mapping

To investigate changes in allele frequencies over time, the GPSM method was used for each pig breed independently. This approach uses a genome-wide linear mixed model framework, with the individual’s generation number as the dependent variable. Thus, the GPSM method models the association between SNP genotypes and generation number, identifying alleles potentially under directional selection (Rowan et al. 2021). The analyses were performed using the GCTA software (Yang et al. 2011), which enabled estimation of SNP effects while accounting for population structure through a genomic relationship matrix. The model used is described as:

$$y=\mu\:+X_ib_i+Zg+e$$

where **y** is a vector of individual generation proxies (i.e., month and year of birth), µ is the sample mean, **X**_i_ is the vector of SNP genotypes for each individual at SNP i, b_i_ is the effect of SNP i, **g** is the vector of random polygenic effects ~$\:N\left(0,\boldsymbol{G}{\sigma\:}_{g}^{2}\right)$, **Z** is the incidence matrix connecting **y** to the random polygenic effects in **g**, **e** is the vector of random residuals ~$\:N\left(0,\boldsymbol{I}{\sigma\:}_{e}^{2}\right)$, **G** is the genomic relationship matrix (created following the first method shown by (VanRaden 2008), $\:{\sigma\:}_{g}^{2}$ is the genomic variance, **I** is an identity matrix, and $\:{\sigma\:}_{e}^{2}$ is the residual variance.

The GPSM analyses were performed using the “-mlma” function in the GCTA software (Yang et al. 2011). Quantile-quantile (Q-Q) plots were generated to evaluate the presence of potential false-positive associations. To control multiple testing, a Bonferroni correction was applied, setting the significance threshold at a 5% family-wise error rate (α = 0.05/total number of SNPs). The SNPs with p-values below this threshold were considered significantly associated with the generation proxy.

Genomic Region Detection and Functional Enrichment Analysis.

To better characterize signatures of past selection and infer the direction of recent or ongoing selection within each breed, gene and QTL annotations were used to interpret the biological functions underlying the genomic regions identified by ROH islands and the GPSM method. The annotations were performed using the R package “GALLO” (Fonseca et al. 2020), based on the latest pig genome reference assembly (Sscrofa11.1; http://useast.ensembl.org/Sus_scrofa/Info/Index) and the Pig QTL Database (Hu et al. 2007). A genomic window of 100 Kb upstream and downstream of each marker was used to retrieve associated genes and QTLs. The identified genes were further analyzed for functional enrichment using the “gprofiler2” package available in R (Reimand et al. 2007).

## Results

### Population structure and genetic diversity metrics

**Fig. 1:**
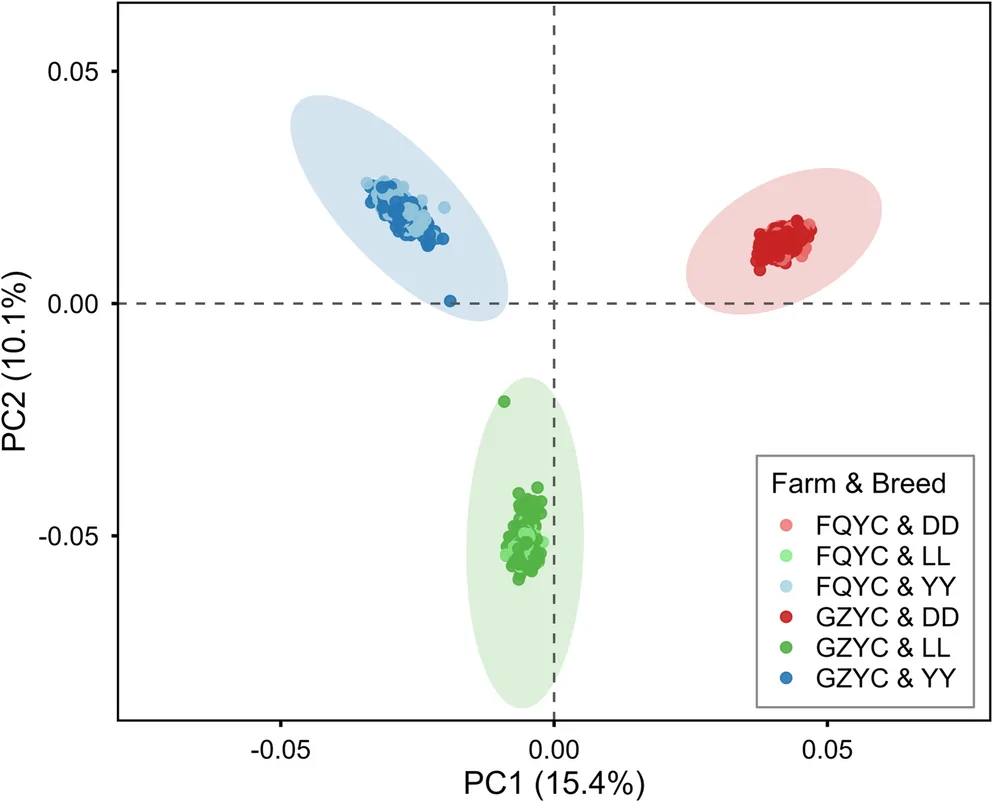
Principal component analysis of the genomic relationship matrix organized by farm and breed. (Abbreviations used in the legend are defined as follows: FQYC = Qingliu Yongcheng farm; GZYC = Guizhou Yongcheng farm; DD = Duroc; LL = Landrace; YY = Yorkshire)

A PCA plot was used to visualize population structure across the three breeds from two farms. The first two components account for 15.4% and 10.1% of the total genetic variation, respectively (Fig. 1). As expected, three main clusters were found and individuals belonging to the same breed consistently grouped together regardless of farm origin, reflecting strong breed-specific genetic structure.

Table 2 summarizes the genetic diversity across the three pig populations. Overall, the diversity metrics indicate comparable levels of within-population genetic variation among DD, LL, and YY. Despite these broadly similar diversity levels, differences in LD suggest distinct population histories, with LL and YY showing lower average LD than DD, consistent with weaker recent selection intensity or larger effective population sizes. In contrast, between population differentiation revealed clearer structure, with DD being more genetically distinct from both LL and YY than LL and YY are from each other, indicating closer genetic relatedness between the latter two populations.

**Table 2:** Genetic diversity metrics in Duroc (DD), Landrace (LL) and Yorkshire (YY)

Genetic distance	DD	LL	YY
	0.71 ± 0.02	0.71 ± 0.02	0.70 ± 0.02
LD	0.34 ± 0.33	0.28 ± 0.29	0.25 ± 0.29
MAF	0.27 ± 0.13	0.28 ± 0.13	0.29 ± 0.13
Observed heterozygosity	0.37 ± 0.02	0.39 ± 0.03	0.38 ± 0.02
Expected heterozygosity	0.36 ± 0.0002	0.37 ± 0.0002	0.38 ± 0.0001

*LD *Linkage disequilibrium (); *MAF* Minor allele frequency; Observed heterozygosity = Proportion of heterozygous genotypes; Expected heterozygosity = 2pq; where p andq are allele frequencies.

### Linkage disequilibrium, effective population size, and consistency of gametic phase

**Fig. 2:**
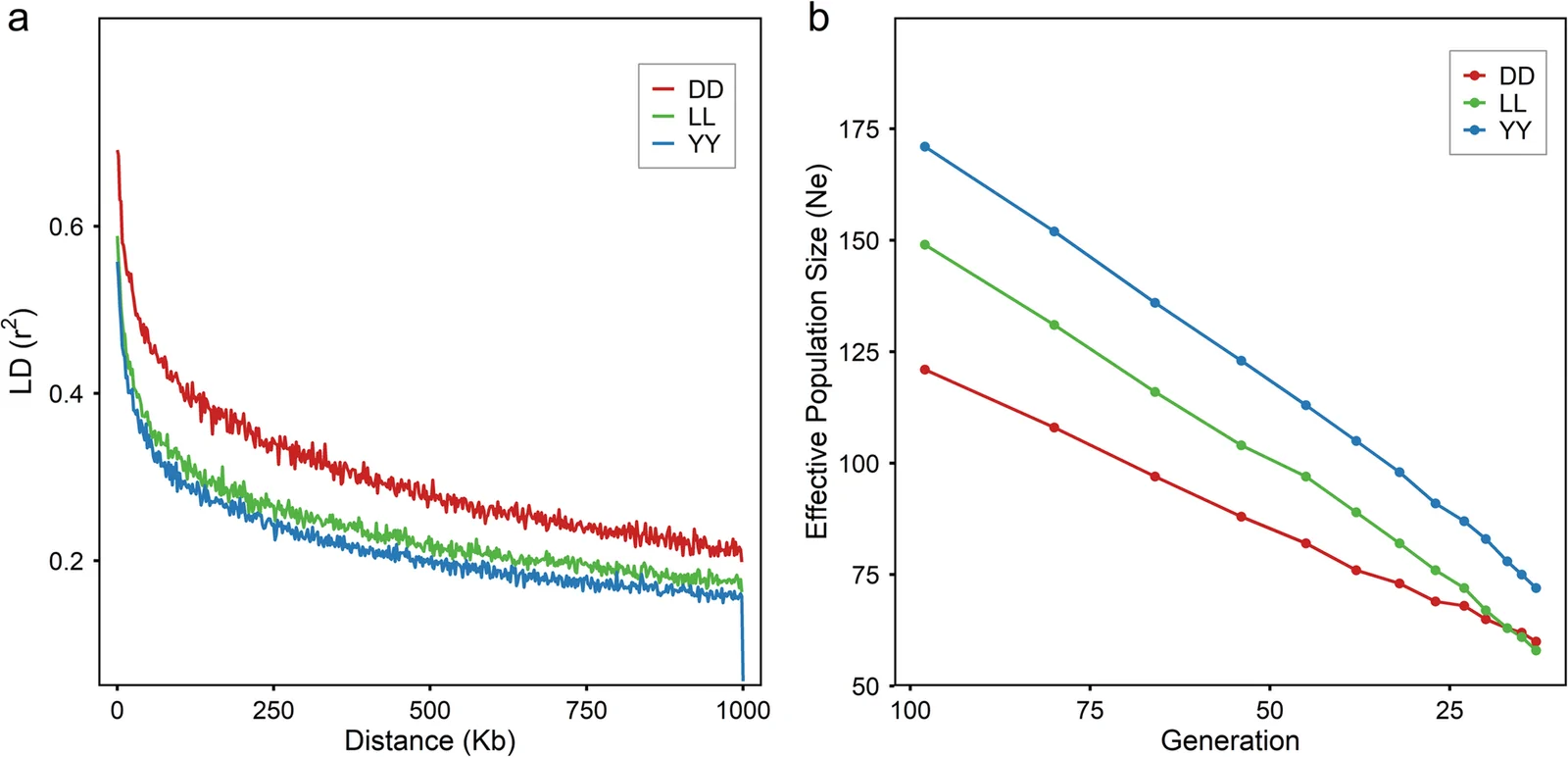
Linkage disequilibrium (LD; **a**) decay and effective population size (Ne; **b**) for Duroc (DD), Landrace (LL), and Yorkshire (YY)

The average LD between adjacent autosomal SNPs was 0.34, 0.28, and 0.25 for DD, LL, and YY, respectively (Table 2). The mean LD values across autosomal chromosomes ranged from 0.29 to 0.41 in DD, 0.22 to 0.33 in LL, and 0.19 to 0.34 in YY. The highest average r² was observed on SSC6 in both DD and LL, and on SSC1 in YY. In contrast, the lowest average r² was detected on SSC11 in DD and YY, and on SSC5 in LL. Overall, DD consistently exhibited the highest levels of LD across chromosomes, followed by LL and YY. The proportions of adjacent SNP pairs with r² ≥ 0.20 and r² ≥ 0.30, along with additional chromosomal LD statistics, are shown in the Supplementary Table S1. The decay of LD with increasing physical distance between autosomal SNPs is shown in Fig. 2A. Across the entire distance range (from 0 to 1,000 Kb), DD consistently exhibited the highest LD across chromosomes, followed by LL and YY.

The Ne of the three breeds was estimated over approximately 13 to 98 past generations (Fig. 2b). Across this period, all populations showed a clear decline in Ne toward recent generations. In the most recent estimates (13 generations ago), Ne was comparatively lower in DD (60) and LL (58) and higher in YY (72). The CGP was also evaluated between breed pairs to assess the feasibility of combining populations into a single training set for SNP effect estimation. Among breed pairs, the CGP are generally low. The highest overall consistency was observed between LL and YY, followed by DD and LL then DD and YY. More details with CGP results are provided in Supplementary Figure S1.

### ROH patterns and inbreeding coefficients

A total of 32,454 ROH were identified in DD (107 ± 8), 21,882 in LL (79 ± 12), and 40,490 in YY (87 ± 10). The average ROH lengths were 6.26 Mb, 6.42 Mb, and 5.70 Mb for DD, LL, and YY, respectively. Compared to the other breeds, YY exhibited the highest number of short ROH (≤ 4 Mb), with 25,066 segments identified (approximately 61.9% of all ROH in this breed), followed by LL with 11,672 segments (53.34%), and then DD with 18,151 (55.93%). The majority of ROH were found in the length range from 2 Mb to 4 Mb among DD, LL, and YY, accounting for 41.35%, 36.58%, and 38.78% of all ROH found in each breed, respectively (Table 3).

**Table 3:** Descriptive statistics of homozygosity in Duroc (DD), Landrace (LL) and Yorkshire (YY)

Average Length (Mb)	Duroc	Landrace	Yorkshire
	6.263	6.421	5.703
**Length Range (Mb)**	1.002–94.907	1.005–194.315	1–247.741
**< 2Mb**	4,732 (14.58%)	3,667 (16.76%)	9,362 (23.12%)
**2-4Mb**	13,419 (41.35%)	8,005 (36.58%)	15,704 (38.78%)
**4-8Mb**	10,429 (32.13%)	5,794 (26.48%)	9,121 (22.53%)
**8-16Mb**	6,276 (19.34%)	2,835 (12.96%)	3,657 (9.03%)
**> 16Mb**	2,330 (7.18%)	1,581 (7.23%)	2,646 (6.53%)
**Total**	32,454 (100%)	21,882 (100%)	40,490 (100%)

Runs of homozygosity (ROH) were classified into five length categories (<2, 2–4, 4–8, 8–16, and >16 Mb). Values represent the total number of ROH detected within each length category, with percentages in parentheses calculated relative to the total number of ROH identified within each breed. Average length and length range refer to the distribution of individual ROH segments observed across all genotyped animals within each breed

**Fig. 3:**
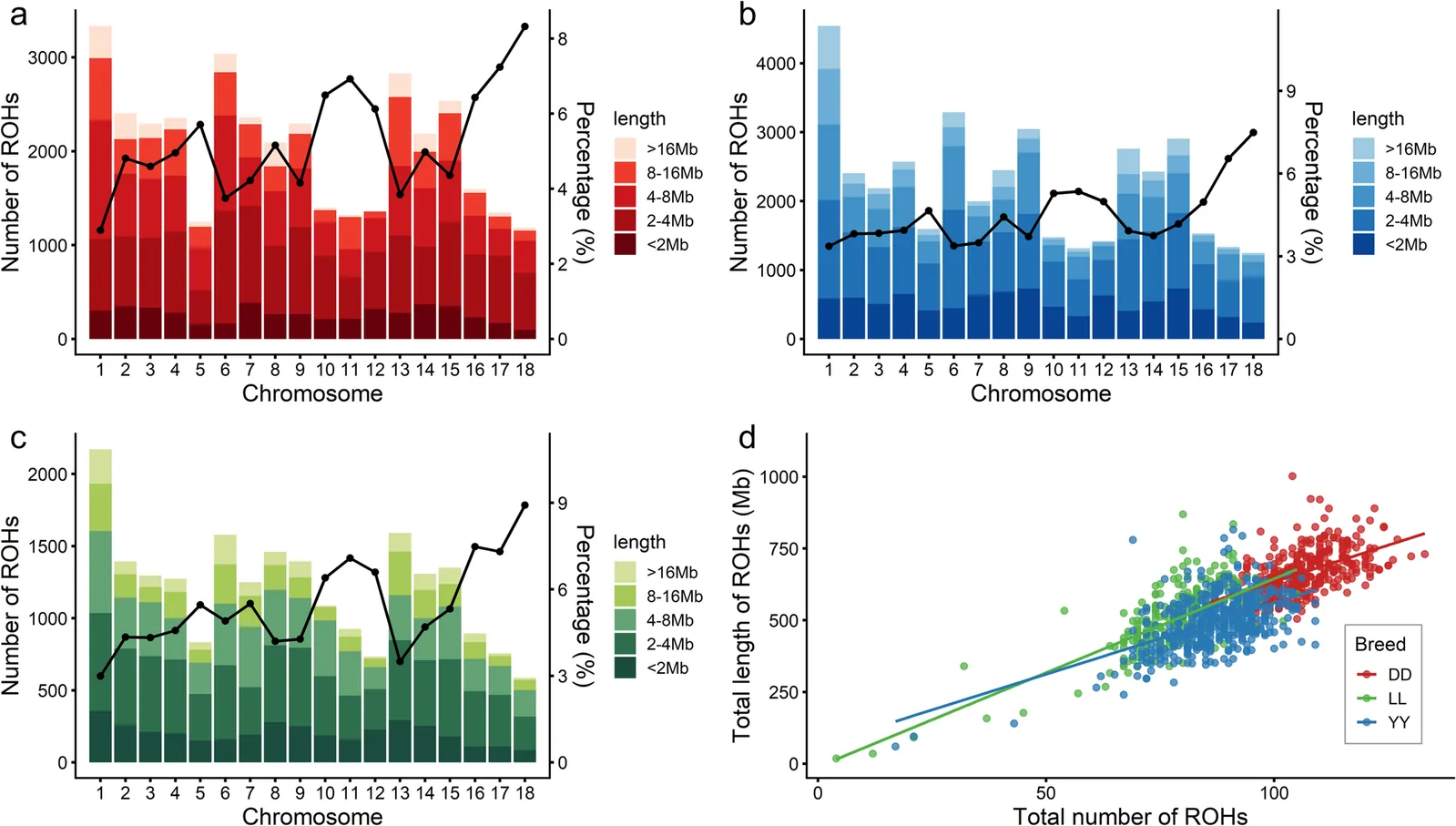
Runs of homozygosity (ROH) distribution across Duroc (DD, **a**), Landrace (LL, **b**) and Yorkshire (YY, **c**). The total number of ROH and length distribution in DD, LL and YY are shown in (**d**)

The ROH distribution pattern is shown in Fig. 3. Overall, the highest and lowest numbers of ROH were observed on SSC1 and SSC18, respectively, for all breeds. In contrast, when measured as a percentage of the chromosome length, the pattern was reversed, the highest value was observed in SSC18 and the lowest was in SSC1 (Fig. 3). As shown in Fig. 3 d, a strong positive correlation was observed between the total length and number of ROH across all breeds: DD (*r* = 0.51, *p* < 0.001), LL (*r* = 0.69, *p* < 0.001), YY (*r* = 0.53, *p* < 0.001).

The inbreeding coefficients estimated using different methods are summarized in Table 4. Across all methods, inbreeding coefficients ranged from − 0.20 to 0.44 in DD, −0.88 to 0.38 in LL, and − 0.70 to 0.36 in YY. Pedigree-based inbreeding showed moderate average values (0.05 in DD, 0.09 in LL, and 0.03 in YY), whereas SNP-based estimators based on homozygosity or genomic relationships (FHOM1, FHOM2, FGRM, and FUNI) were centered closer to zero and displayed wider ranges. FROH consistently showed the highest inbreeding levels across the three breeds, with mean values of 0.29 in DD, 0.22 in LL, and 0.22 in YY. Partitioning FROH by ROH length indicated that longer ROH segments (≥ 8 Mb) contributed substantially to total inbreeding in all breeds, while shorter ROH (< 4 Mb) accounted for smaller proportions.

**Table 4:** Summary of inbreeding coefficient metrics across Duroc (DD), Landrace (LL) and Yorkshire (YY)

Breed	Metric	*n*	min	max	mean	sd
DD	FPED	348	0	0.12	0.05	0.02
	FHOM1	348	−0.14	0.28	−0.01	0.06
	FGRM	348	−0.2	0.22	−0.01	0.07
	FHOM2	348	−0.17	0.3	−0.01	0.06
	FUNI	348	−0.11	0.21	−0.01	0.04
	FROH	348	0	0.44	0.29	0.04
	< 2 Mb	348	0	0.02	0.01	0
	2–4 Mb	348	0	0.07	0.05	0.01
	4–8 Mb	348	0	0.11	0.07	0.01
	8–16 Mb	348	0	0.16	0.09	0.02
	> 16 Mb	348	0	0.24	0.07	0.03
LL	FPED	276	0	0.17	0.09	0.03
	FHOM1	276	−0.75	0.15	−0.03	0.09
	FGRM	276	−0.38	0.18	−0.03	0.08
	FHOM2	276	−0.88	0.17	−0.03	0.1
	FUNI	276	−0.54	0.09	−0.03	0.07
	FROH	276	0.01	0.38	0.22	0.05
	< 2 Mb	276	0	0.02	0.01	0
	2–4 Mb	276	0	0.06	0.04	0.01
	4–8 Mb	276	0	0.11	0.05	0.01
	8–16 Mb	276	0	0.1	0.05	0.02
	> 16 Mb	276	0	0.24	0.08	0.04
YY	FPED	464	0	0.28	0.03	0.02
	FHOM1	464	−0.7	0.18	−0.02	0.06
	FGRM	464	−0.32	0.17	−0.02	0.06
	FHOM2	464	−0.7	0.18	−0.02	0.07
	FUNI	464	−0.51	0.15	−0.02	0.05
	FROH	464	0	0.36	0.22	0.04
	< 2 Mb	464	0	0.03	0.01	0
	2–4 Mb	464	0	0.06	0.04	0.01
	4–8 Mb	464	0	0.08	0.05	0.01
	8–16 Mb	464	0	0.09	0.04	0.01
	> 16 Mb	464	0	0.24	0.08	0.04

n, number of individuals; min, minimum individual inbreeding coefficient; max, maximum individual inbreeding coefficient; mean, average inbreeding coefficient; sd, standard deviation. Metric, the different methods were used to calculate inbreeding. The inbreeding metrics are: FPED, pedigree based inbreeding coefficient calculated using the pedigree information; FGRM, genomic inbreeding coefficient based on the additive genomic relationship matrix; FHOM1, genomic inbreeding coefficient based on the difference between observed and expected homozygosity; FHOM2, genomic inbreeding coefficient based on the proportion of homozygous genotypes; FUNI, genomic inbreeding coefficient based on the correlation between uniting gametes. FROH, runs of homozygosity based inbreeding coefficient calculated as the total length of ROHs divided by the total length of the autosomal genome. FROH was further partitioned into ROH length classes: <2 Mb, 2–4 Mb, 4–8 Mb, 8–16 Mb, and >16 Mb

**Fig. 4:**
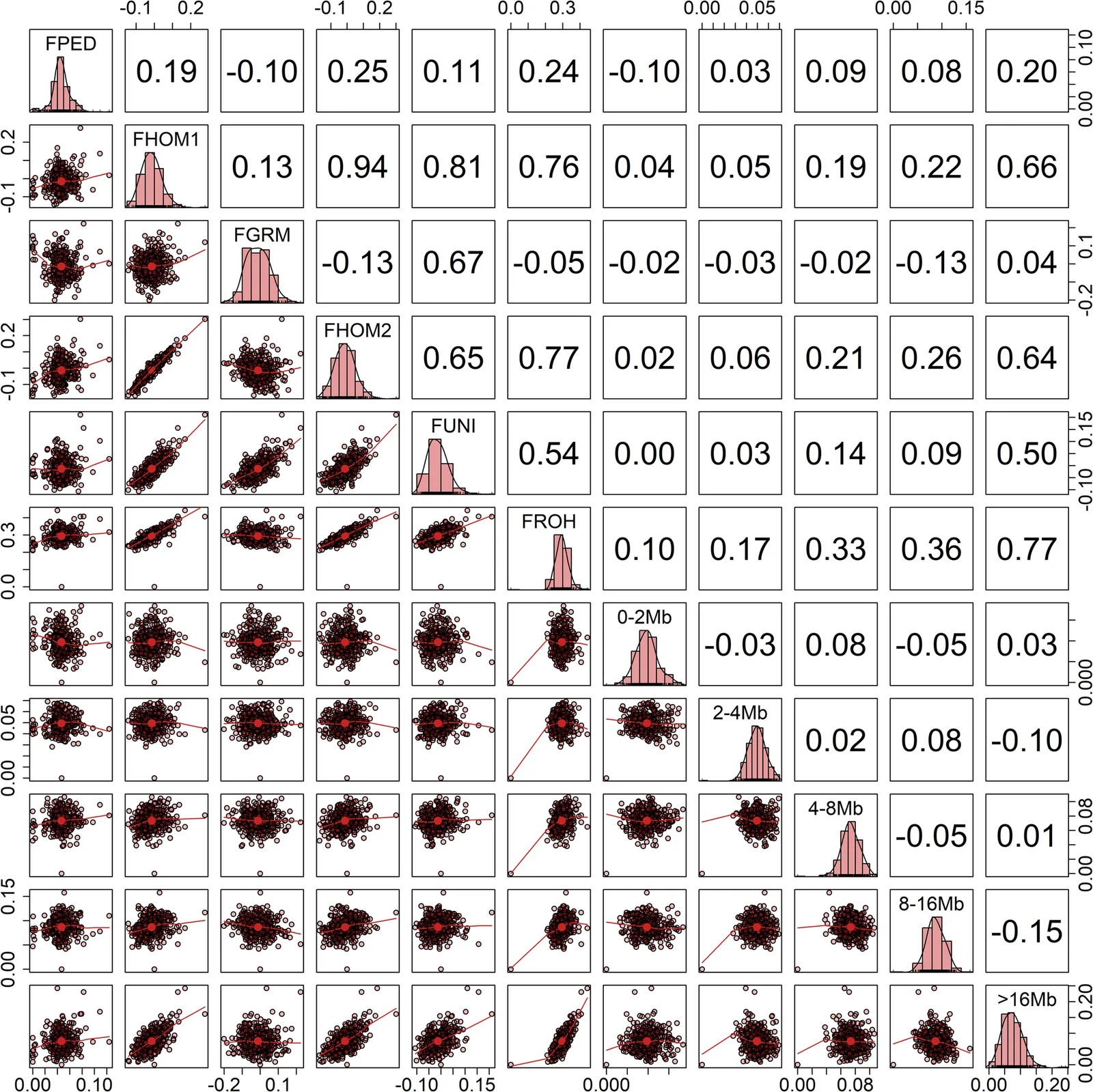
Scatterplots, distributions, and correlations of genomic and pedigree inbreeding coefficients in Duroc (DD)

**Fig. 5:**
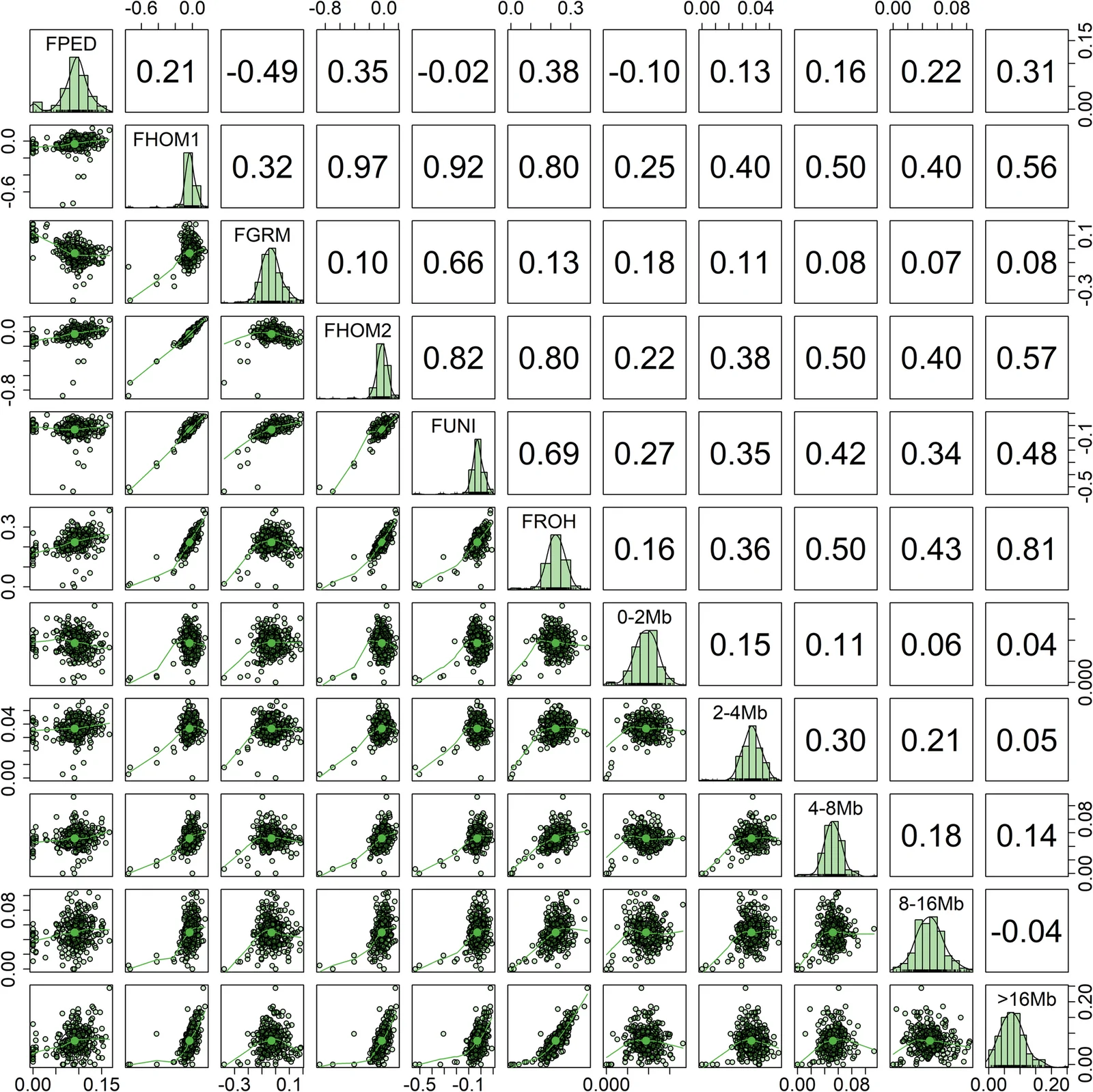
Scatterplots, distributions, and correlations of genomic and pedigree inbreeding coefficients in Landrace (LL)

**Fig. 6:**
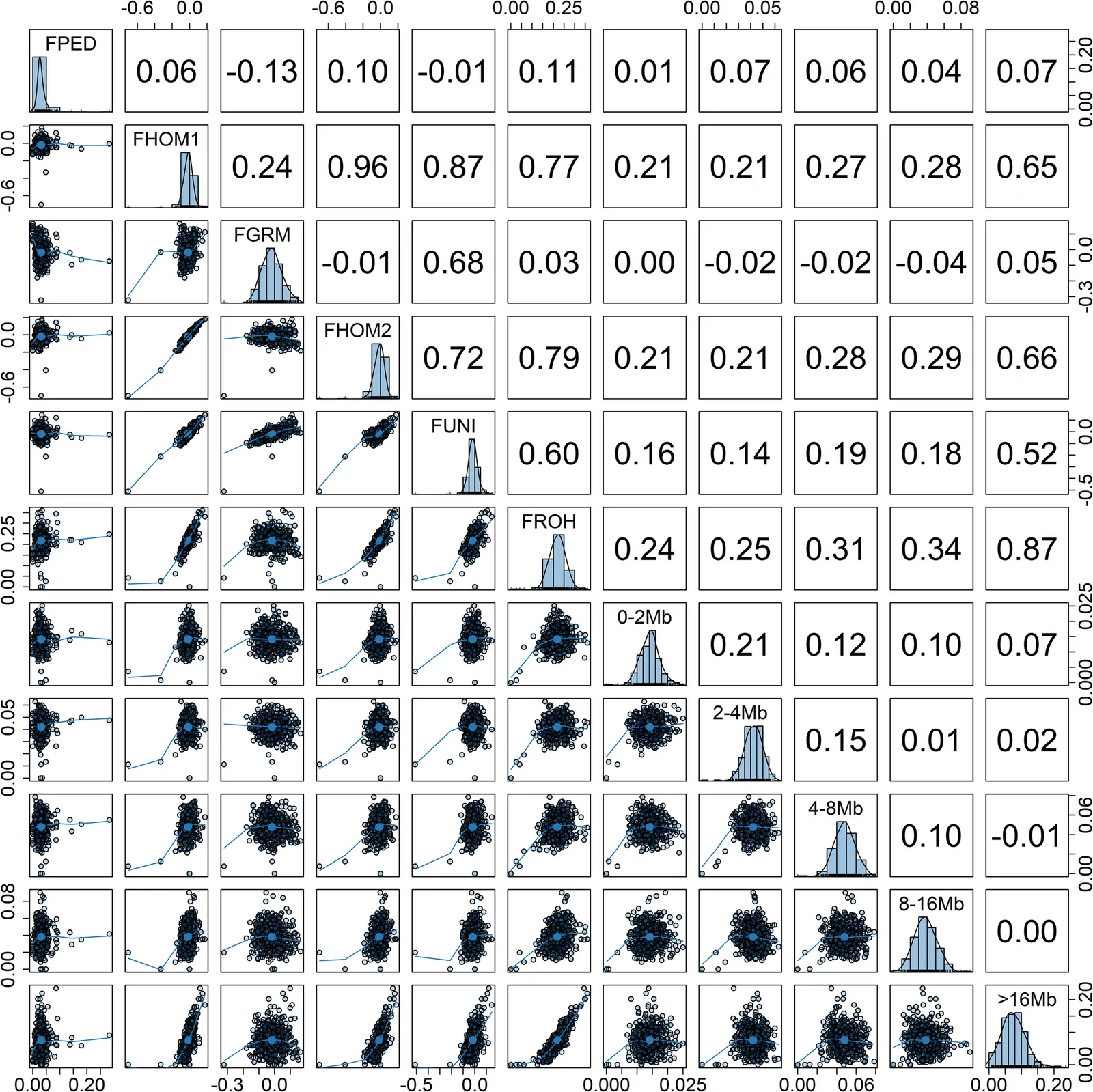
Scatterplots, distributions, and correlations of genomic and pedigree inbreeding coefficients in Yorkshire (YY)

The correlations among inbreeding coefficients within the DD, LL, and YY populations are shown in Figs. 4, 5 and 6. Among all measures (Figs. 4, 5 and 6), FROH consistently showed the highest inbreeding estimates across the three pig breeds. In DD pigs, the highest values were observed for FHOM1 (0.278), FGRM (0.223), FHOM2 (0.301), and FUNI (0.211). Notably, the maximum FROH value (0.442) was also observed for DD, reflecting substantial homozygosity as captured by ROH-based metrics. In LL pigs, the highest values for FHOM1 (0.151), FGRM (0.183), FHOM2 (0.168), and FUNI (0.088) were lower than those in DD, with the highest FROH reaching 0.384. In YY pigs, the maximum inbreeding estimates for FHOM1 (0.176), FGRM (0.165), FHOM2 (0.180), and FUNI (0.146) were moderate relative to the other two breeds, and the highest FROH was 0.360.

High correlations (> 0.81) were observed consistently among FHOM1 and FHOM2, and among FHOM1 and FUNI across all three pig populations. In the DD population (Fig. 4), moderate to strong correlations were observed among the homozygosity-based genomic metrics. For instance, FHOM1 and FHOM2 were highly correlated (*r* = 0.94), and both were strongly associated with FROH (*r* = 0.76–0.77). The FUNI also showed a high correlation with FHOM1 (*r* = 0.81) and FHOM2 (*r* = 0.65), but only a moderate correlation with FROH (*r* = 0.54). Pedigree-based inbreeding exhibited a moderate correlation with FHOM2 and FROH (*r* = 0.25 and 0.24, respectively), but weak or negative correlations with FGRM and short FROH categories (*r* = − 0.10 with < 2 Mb). FGRM displayed only weak or negative correlations with other inbreeding metrics.

In LL (Fig. 5), similar trends were observed. FHOM1 and FHOM2 were again highly correlated (*r* = 0.97), and both showed strong correlations with FROH (*r* = 0.84). FUNI was also strongly correlated with FHOM1 (*r* = 0.92), FHOM2 (*r* = 0.82), and moderately with FROH (*r* = 0.72). Pedigree inbreeding was moderately correlated with FHOM2 (*r* = 0.35) and FROH (*r* = 0.39) but negatively associated with FGRM (*r* = −0.49). Compared to DD, LL exhibited stronger positive correlations between short FROH categories (< 2 Mb and 2–4 Mb) and homozygosity-based coefficients.

For YY pigs (Fig. 6), the correlation patterns were close like those observed in DD and LL, with strong internal consistency among FHOM1, FHOM2, FUNI, and FROH. Pedigree inbreeding continued to show only modest correlation with genomic estimates, while FGRM tended to be weakly correlated or even negatively associated with other inbreeding measures.

### ROH islands and GPSM

**Fig. 7:**
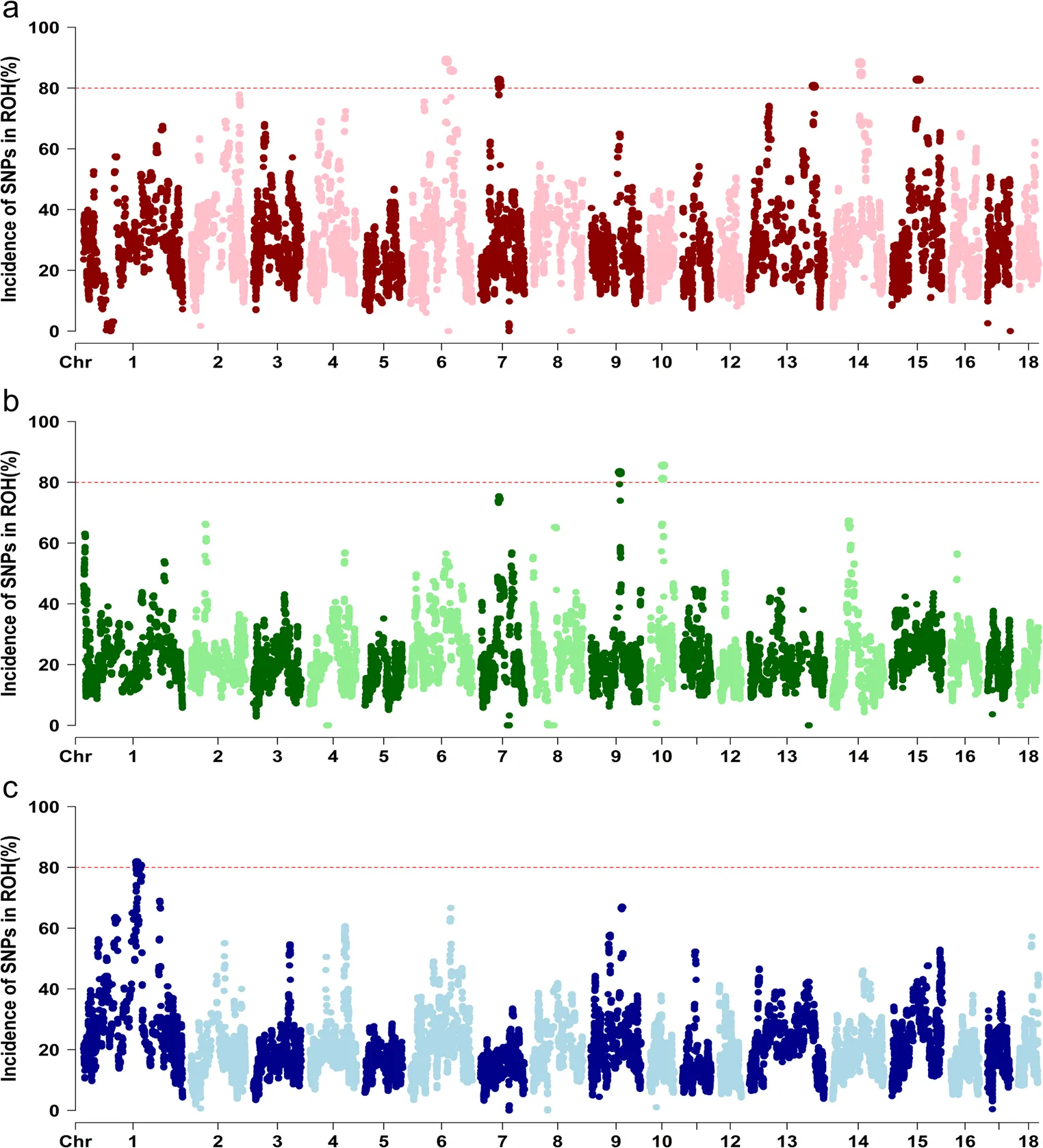
Manhattan plot of the occurrence (%) of SNPs in runs of homozygosity in Duroc (DD, **a**), Landrace (LL, **b**) and Yorkshire (YY, **c**)

The proportion of occurrence of SNPs within a ROH in DD, LL, and YY are shown in Fig. 7. The ROH islands and significant SNPs were identified across the three breeds (Supplementary Table 3). Specifically, markers located within ROH regions shared by at least 80% of individuals in each population were defined as ROH islands, corresponding to the top 0.79% in DD pigs, the top 0.16% in LL pigs, and the top 0.18% in YY pigs.

In DD, six ROH islands were identified, ranging from 2.73 to 5.02 Mb in length and encompassing 390 SNPs. Within the 100-kb flanking regions of these islands, a total of 254 genes and 115 QTLs were annotated. These QTLs were mainly associated with production, reproduction, and meat and carcass traits. In LL, two ROH islands were detected, each spanning approximately 2.0 Mb and comprising 51 and 52 SNPs, respectively. A total of 75 genes and 10 QTLs were annotated within these regions, primarily related to reproduction and meat and carcass traits. In YY, two ROH islands were identified with lengths of 2.35 Mb and 3.93 Mb, comprising 48 and 64 SNPs, respectively. Within these regions, 58 genes and 68 QTLs were annotated, predominantly associated with production, reproduction, and meat and carcass traits. Detailed information on genes, QTLs, and gene ontology enrichment for ROH islands across DD, LL, and YY is provided in Supplementary Tables S3–S5.

**Fig. 8:**
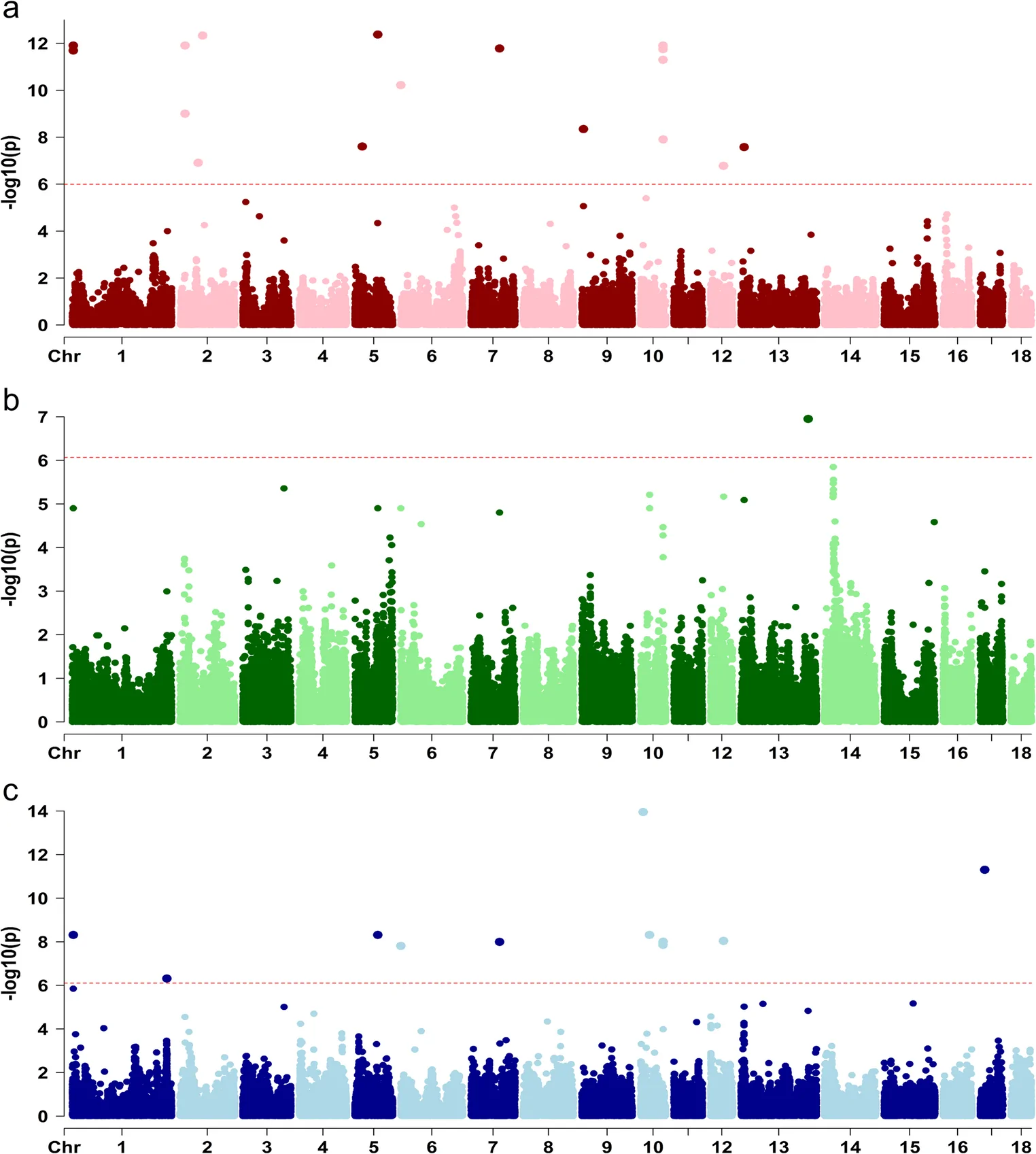
Manhattan plot of Generation Proxy Selection Mapping (GPSM) among Duroc (DD, **a**), Landrace (LL, **b**) and Yorkshire (YY, **b**)

Figure 8 shows the results of the GPSM analysis, highlighting genomic regions potentially undergoing recent selection across the three breeds. In DD, six GPSM loci were identified across multiple autosomes. Within the annotated regions surrounding these significant SNPs, 10 genes were detected. The corresponding QTLs were mainly associated with production, reproduction, and meat and carcass traits. Only one marker was detected in LL. This locus overlapped a single annotated gene (*NCAM2*) and a limited number of QTLs, which were primarily related to meat and carcass traits. In YY, six markers were identified across several autosomes. Within these regions, 12 genes were annotated. The associated QTLs were also predominantly related to production, reproduction, and meat and carcass traits. Additional details on GPSM-based gene and QTL annotations are provided in the Supplementary Tables S6–S8.

### Shared and breed-specific genes and QTLs

**Fig. 9:**
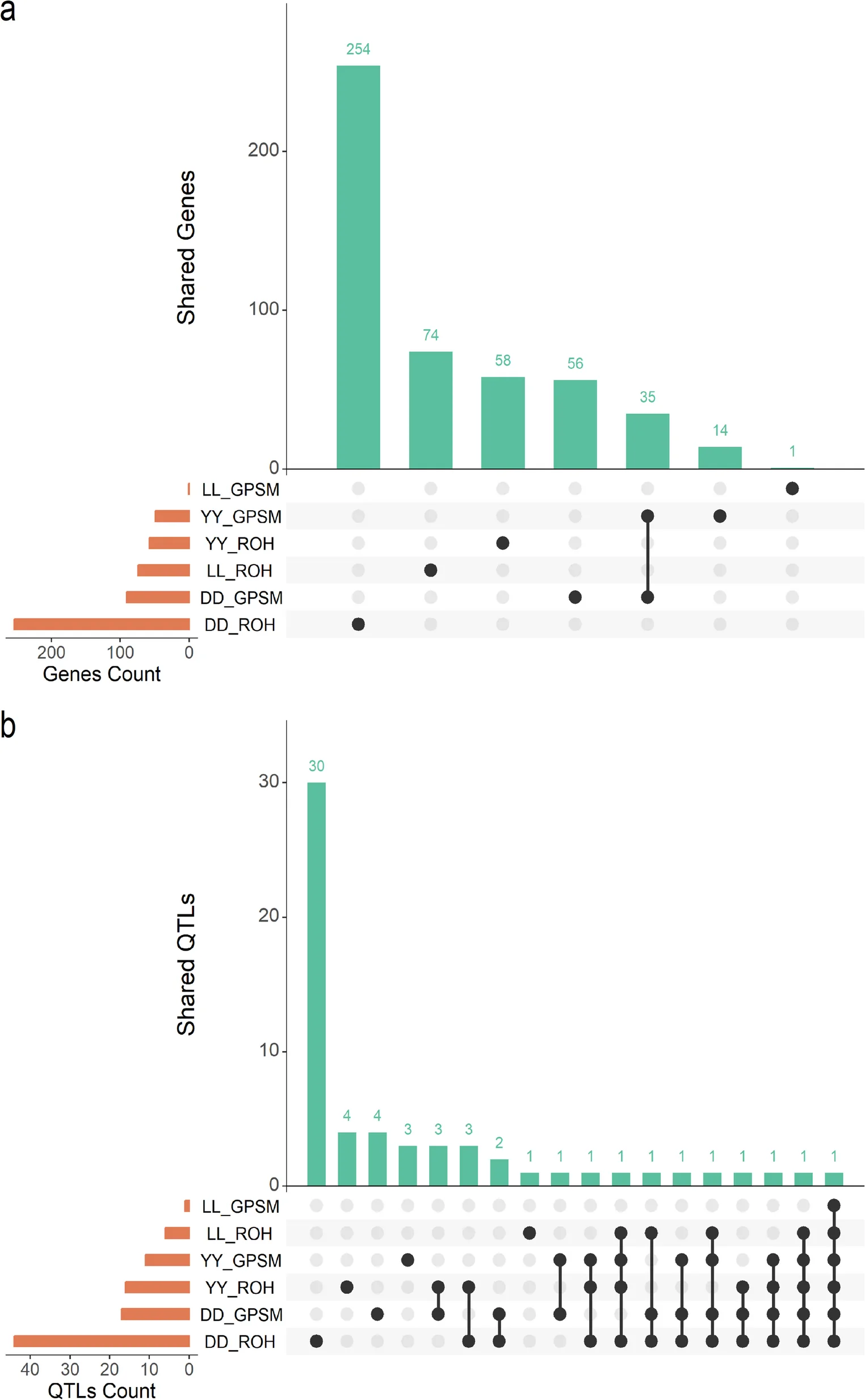
Comparative analysis of genes (**a**) and QTLs (**b**) identified by the Generation Proxy Selection Mapping (GPSM) and runs of homozygosity (ROH) across Duroc (DD), Landrace (LL), and Yorkshire (YY)

Both GPSM and ROH island analyses identified genomic regions potentially under selection, but their overlap in annotated genes and QTLs was generally low. Moreover, no significantly enriched GO terms were detected in the LL and YY, as detailed in Supplementary Tables S6–S8. Figure 9 shows the shared and specific genes and QTLs across DD, LL and YY, based on the ROH island and GPSM method.

Based on ROH island analysis, no genes were found to be shared between any two breeds. However, three QTL-associated traits were identified as shared across all three pig breeds: offspring number, subcutaneous fat thickness, and longissimus muscle depth. Additionally, three traits were shared between DD and LL (i.e., PRRSV susceptibility, semen odor), and six traits between DD and YY (i.e., marbling, vertebra number, and birth weight variability). No shared traits were detected between LL and YY under the ROH-based approach. Breed-specific results revealed that 254, 74, and 58 genes were uniquely identified in DD, LL, and YY, respectively. The number of breed-specific QTL-associated traits was 33 in DD, 7 in YY, and none in LL. These breed-specific traits from DD and YY were mainly related to meat quality (i.e., marbling, pH, dressing percentage), reproduction (i.e., maternal infanticide, weaning-to-estrus interval), and health-related traits (i.e., white and red blood cell counts, PRRSV susceptibility).

Based on GPSM analysis, 35 genes were found to be shared between DD and YY, while no shared genes were detected between any other breed pairs. One QTL-associated trait, longissimus muscle depth, was identified across all three breeds. Additionally, five traits were shared between DD and YY: subcutaneous fat thickness, teat number, intramuscular fat content, cooking loss, and maternal infanticide. Breed-specific results revealed 56 genes uniquely identified in DD, 14 in YY, and only 1 in LL. The number of breed-specific QTL-associated traits was 11 in DD and 5 in YY, with none identified in LL. These breed-specific traits in DD were primarily related to reproduction and health (i.e., number of functional sperm, semen odor, boar sexuality score), growth (i.e., days to 100 kg, body condition score), and hematological parameters (i.e., LDL cholesterol, mean corpuscular volume, red cell distribution width). In YY, the breed-specific traits included growth and development (i.e., average daily gain, cannon bone circumference), reproduction (i.e., offspring number, sperm abnormality rate), and health traits (i.e., platelet count).

## Discussion

### Current genetic diversity

In the study a clear cluster of the DD, LL and YY was observed in PCA plot, reflecting substantial genetic differentiation among the breeds. This pattern aligns with their unique breeding histories and roles in commercial production systems. In three-way crossbreeding systems, DD is typically used as a terminal sire line and has undergone intense selection for growth and carcass traits (Alonso et al. 2009), likely contributing to its distinct clustering. In contrast, LL and YY, both maternal lines, clustered more closely together, suggesting shared selection pressures related to reproductive performance and maternal ability (Weller, 2016). Consistent with the PCA results, LD patterns reveal breed-specific genetic structures. Average LD (r²) between adjacent SNPs was higher in DD (0.34), followed by LL (0.28), and YY (0.25). DD exhibited slower LD decay, which is indicative of stronger historical selection. In contrast, the faster LD decay observed in LL and YY suggests larger historical Ne and greater genetic diversity. These findings are in line with previous studies. For example, Badke et al. (Badke et al. 2012) reported similar LD decay patterns across US breeds using the PorcineSNP60 BeadChip. Similarly, Ai et al. (Ai et al. 2013) found elevated LD in Western commercial pigs relative to Chinese indigenous breeds. Complementing these results, pairwise FST estimates confirmed DD’s greater genetic divergence (0.34 with LL, 0.33 with YY), compared to the closer relationship between LL and YY (FST = 0.22). These values further support DD’s distinct evolutionary trajectory under directional selection for terminal sire characteristics.

In addition to between breed comparisons, breed genetic diversity was evaluated using DST, MAF, and heterozygosity metrics. The average pairwise genetic distance (DST) was slightly higher in DD and LL compared to YY, suggesting comparable levels of allelic variation within populations (Hayah et al. 2023). Similarly, the average MAF was broadly consistent across breeds, indicating a balanced representation of common and rare alleles in the datasets. Heterozygosity estimates provided further insights into within-population variability (Hayah et al. 2023). Observed heterozygosity (HO) ranged from 0.37 in DD to 0.39 in LL, while expected heterozygosity (HE) values were nearly identical among breeds, ranging from 0.36 to 0.38. The relatively high and comparable levels of HO and HE across breeds suggest that, despite selection pressure, substantial genetic variation is maintained within each population, likely due to the use of structured breeding programs and genomic selection strategies aimed at preserving diversity while improving targeted traits. Overall, these results indicate that while DD is more genetically distinct from the maternal breeds, all three populations retain moderate to high levels of within-breed diversity, which is essential for long-term genetic improvement and population sustainability (Zhao et al. 2019).

### ROH structure and inbreeding coefficients

The formation of ROH patterns is largely influenced by historical population bottlenecks, inbreeding, genetic drift, natural and long-term artificial selection (Ceballos et al. 2018). Therefore, analyzing ROH patterns across breeds can provide valuable information on the relative timing of autozygosity events. Specifically, longer ROH was generally attributed to more recent common ancestry, whereas shorter ROH tend to reflect more ancient demographic processes (Bosse et al. 2012; Dixit et al. 2020). In this study, DD exhibited the highest number of long ROH (> 16 Mb), consistent with more recent inbreeding events likely driven by strong directional selection within a relatively small Ne. In contrast, YY showed a greater abundance of short ROH (< 4 Mb), which are typically associated with more ancient inbreeding or a historically large and diverse breeding base. LL showed an intermediate ROH pattern, characterized by fewer segments overall but the longest average ROH length (6.42 Mb), suggesting a mix of past demographic constriction and moderate recent inbreeding. These different breeds ROH patterns suggest that while all three breeds have undergone varying degrees of selection and inbreeding, the timing and intensity of these processes differ among them. The predominance of longer ROH in DD might indicate more recent bottlenecks or intensive selection, while the shorter ROH in YY suggests a more diverse historical foundation with ancient inbreeding events. LL, located between these extremes, appears to have undergone a comparatively balanced process of selection and genetic management.

Correlations between pedigree-based (FPED) and genomic-based inbreeding estimates were generally weak. Although all inbreeding metrics aim to quantify relatedness within a population, they differ fundamentally in their conceptual definitions, statistical properties, and the genomic information they emphasize, which together explain the highly diverse correlations observed among metrics in Figs. 4, 5 and 6. Overall, FGRM, FHOM1, and FHOM2 are inherently sensitive to allele frequency shifts driven by genetic drift and directional selection. Especially FGRM in closed and intensively selected livestock populations, such shifts can produce low correlations, even negative ones (Villanueva et al. 2021). The FUNI metric, which follows Wright’s definition (Wright 1922), can capture variation in inbreeding arising from distant ancestors (Kardos et al. 2018). However, its performance is optimized for large populations and may be less suitable for finite and highly structured livestock populations (Polak et al. 2021). The FROH metric fundamentally differs from the others because it directly quantifies realized autozygosity as the proportion of the genome contained within runs of homozygosity, independent of allele frequency assumptions (McQuillan et al. 2008). For this reason, FROH has become one of the most widely used measures of genomic inbreeding (Keller et al. 2012). Consistent with previous research, the correlation between pedigree-based (FPED) and genomic-based inbreeding estimates was generally weak. For instance, (Lopes et al. 2013) reported low correlations across Duroc, Yorkshire, and Pietrain lines, a trend later mirrored in Landrace and Yorkshire populations (Zanella et al. 2016). In the present study, FROH values were consistently higher than FPED across all breeds, highlighting the presence of unrecorded or inbreeding that pedigrees often fail to capture. Similar disparities have been documented in both commercial and autochthonous Italian pigs, where FROH systematically exceeded FPED due to incomplete pedigree depth or complex founder histories (Schiavo et al. 2020). Furthermore, the correlation between FROH and FPED varies substantially in the literature. While some studies report negligible correlations (e.g., 0.04 in Landrace and Large White; (Joaquim et al. 2019), others have found values as high as 0.60 in Iberian and commercial breeds (Saura et al. 2015; Schiavo et al. 2020). Our results range from 0.38 in Landrace to 0.11 in Yorkshire. Notably, this correlation strengthened as the minimum ROH length threshold increased. This pattern suggests that FPED primarily captures recent inbreeding manifested as long ROH segments, whereas shorter ROH, reflecting more ancient autozygosity, remains largely unaccounted for by pedigree data.

###  Genomic regions and genes subject to selection

The ROH islands are widely used to characterize population genomic footprints of autozygosity and the long-term effects of demographic history and historical selection (Ceballos et al. 2018; Mulim et al. 2022). In the present study, no shared genes were detected among the ROH islands identified in the DD, LL, and YY, indicating that these ROH signals predominantly reflect breed-specific long-term selection trajectories and demographic processes. This finding is highly consistent with previous studies. For example, (Tang et al. 2020) reported largely non-overlapping genomic regions under selection among DD, LL, and YY, reflecting distinct breed histories and selection pathways. Moreover, even within the same breed, independent breeding programs may leave distinct genomic footprints of selection (Bertolini et al. 2024). Also, from a biological perspective, such breed specificity is expected, as three-way crossbreeding system were broadly used to produce commercial pigs (Weller, 2016), i.e.: DD has long been selected as a terminal sire line with emphasis on growth rate and carcass traits, whereas LL and YY have been more strongly shaped by selection for maternal and reproductive performance. Importantly, the breed-specific selection patterns revealed by ROH islands do not imply the absence of shared selection constraints among breeds in modern commercial breeding systems. Unlike ROH analyses, which primarily capture historical processes, generation proxy selection mapping (GPSM) is designed to detect ongoing or recent selection by tracking subtle allele frequency changes across generations (Grohmann et al. 2023). Accordingly, although GPSM identified fewer significant loci and annotated genes than ROH-based analyses, the associated QTLs were predominantly related to economically important traits such as feed efficiency, muscle development, reproduction, and health, consistent with its ability to capture contemporary selection dynamics in commercial populations.

While direct overlap between ROH and GPSM-implicated genes was limited, partial convergence was evident across multiple biological levels. At the gene level, for instance, the partial overlap of GPSM-implicated genes between DD and YY, despite their clear separation in PCA, suggests independent polygenic selection acting on similar biological functions across divergent genomic backgrounds. At the QTL level, this functional convergence was further corroborated, where multiple loci associated with subcutaneous fat thickness, longissimus muscle depth, litter size, and teat number were jointly identified across breeds. This pattern suggests that, although the specific genomic regions and genes responding to selection may differ among breeds, selection may act on similar biological functions related to economically relevant traits. Similar patterns have been observed in several studies, where breed-specific genomic regions under selection are associated with analogous production and reproductive traits, indicating functional or trait-level convergence despite distinct genetic backgrounds and selection histories (Mekonnen et al. 2024; Wang et al. 2017).

Notably, in contrast to genome-wide association studies, which explicitly model phenotypic variation, ROH and GPSM approaches are designed to identify genomic regions under past and ongoing selection, along with their potential functional relevance (Decker et al. 2012; Rowan et al. 2021). Consequently, these methods provide insights into which genomic regions may have been subject to selection and the biological functions they may be associated with. Gene and QTL annotations in this study reflect the biological functions and traits potentially targeted by past and ongoing selection, as genomic regions identified through allele frequency shifts serve as indicators of the underlying functional processes.

Overall, breed-specific ROH islands reflecting historical selection, together with the partially overlapping trait-level signals identified by GPSM, provide complementary insights into selection processes operating across distinct temporal and genetic scales. The ROH-based analyses are particularly informative for characterizing long-term inbreeding accumulation and historical selection footprints resulting from past demographic events and sustained selection pressures (Ceballos et al. 2018; McQuillan et al. 2008). In contrast, GPSM is specifically designed to detect dynamic allele frequency changes driven by ongoing selection in modern breeding populations (Rowan et al. 2021). Integrating these two approaches, therefore, offers a more comprehensive framework for understanding how selection shapes genomic architecture over time and how distinct genetic processes may ultimately converge in commercial pig populations.

### Limitations and future directions

While a previous study demonstrated that GPSM can effectively detect polygenic selection signals in commercial pig populations, even over relatively short time scales of approximately 4 to 10 years (Grohmann et al. 2023), the genotyped individuals in the present study were born between 2020 and 2024, lying at the lower boundary of this temporal range and thus representing a potential limitation. Consequently, the identified loci should be understood with caution as reflecting recent allele frequency shifts rather than established long-term selection patterns. Future studies should try to expand the reference population across additional generations and incorporate higher-density genomic and phenotypic data to improve robustness of selection mapping.

## Conclusion

This study characterized population structure, genetic diversity, and demographic patterns in DD, LL, and YY from a long-term closed commercial breeding system, revealing clear breed-specific genomic architectures consistent with their distinct breeding objectives. Analyses of inbreeding and runs of homozygosity further highlighted the value of genomic data, demonstrating that genomic information may substantially improve the accuracy of inbreeding estimates. By applying generation proxy selection mapping, genomic regions undergoing recent allele frequency changes were identified, complementing ROH-based signals of historical selection. Functional annotation of regions jointly identified by ROH islands and GPSM indicated that, despite limited overlap at the gene level, selection across breeds partially converges on similar economically important traits. Our findings contribute to a deeper understanding of the genomic consequences of long-term closed breeding and provide reference information to support consideration of breeding strategies that balance continued selection for productivity with the maintenance of genetic diversity in modern commercial pig populations.

## Supplementary Information

Below is the link to the electronic supplementary material.


Supplementary Material 1



Supplementary Material 2


## Data Availability

The datasets generated or analyzed during the current study are not publicly available due to commercial confidentiality. Access to the data may be granted upon reasonable request, subject to approval by the Yongcheng BREEDING Group Co., Ltd.
