# Supplementary material for: Genomic diversity, inbreeding, and selection signatures in duroc, landrace, and yorkshire pigs from a long-term closed breeding system
Source: J Appl Genet. 2026 Jun 3;67(3):769–86. doi: 10.1007/s13353-026-01072-9 (PMC13534200; doi:10.1007/s13353-026-01072-9)

Supplementary_Figure_S1: Consistency of Gametic Phase Across Duroc, Landrace and Yorkshire

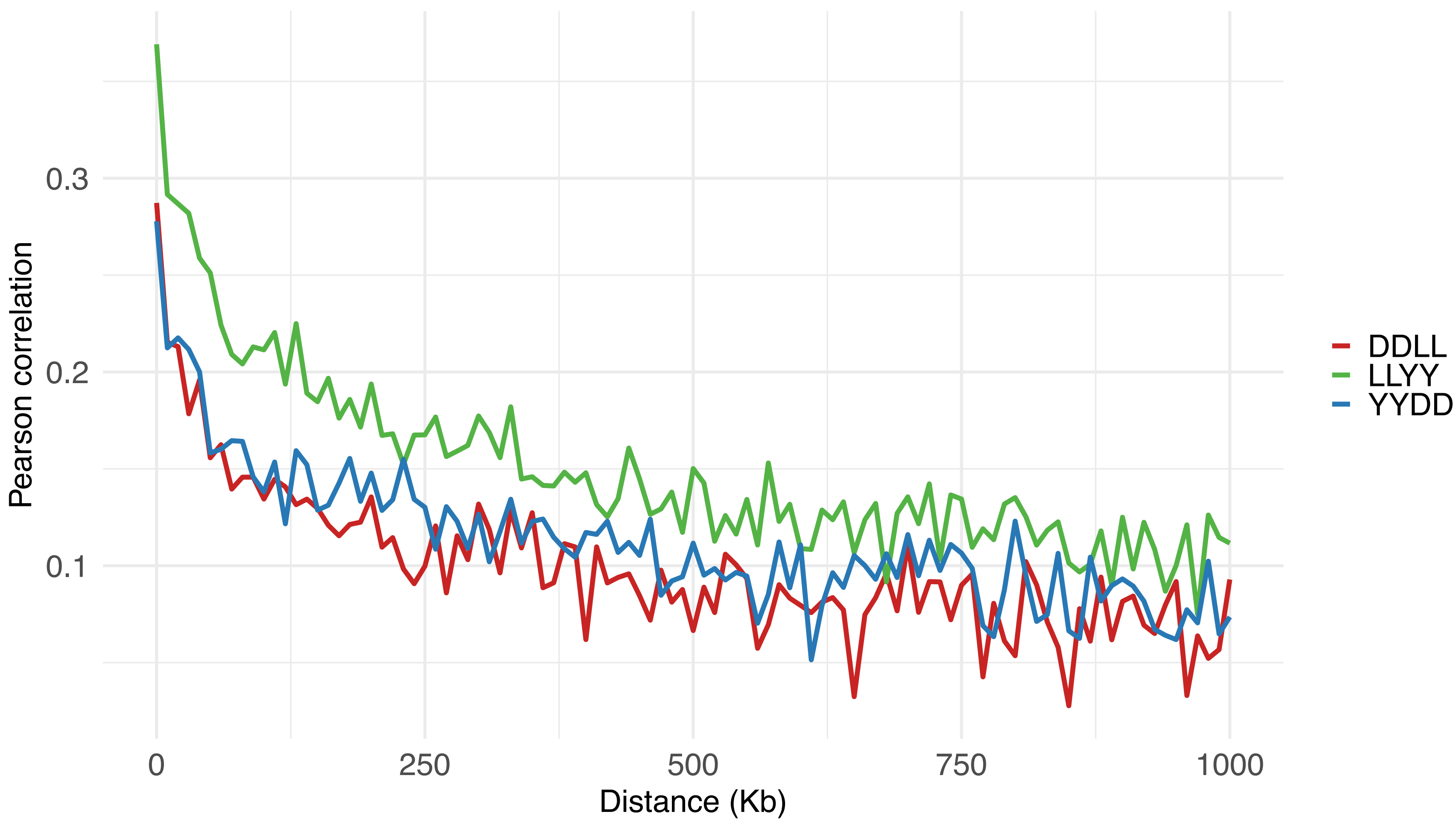

Supplementary_Figure_S2: Number of Shared Genes Across Duroc, Landrace and Yorkshire

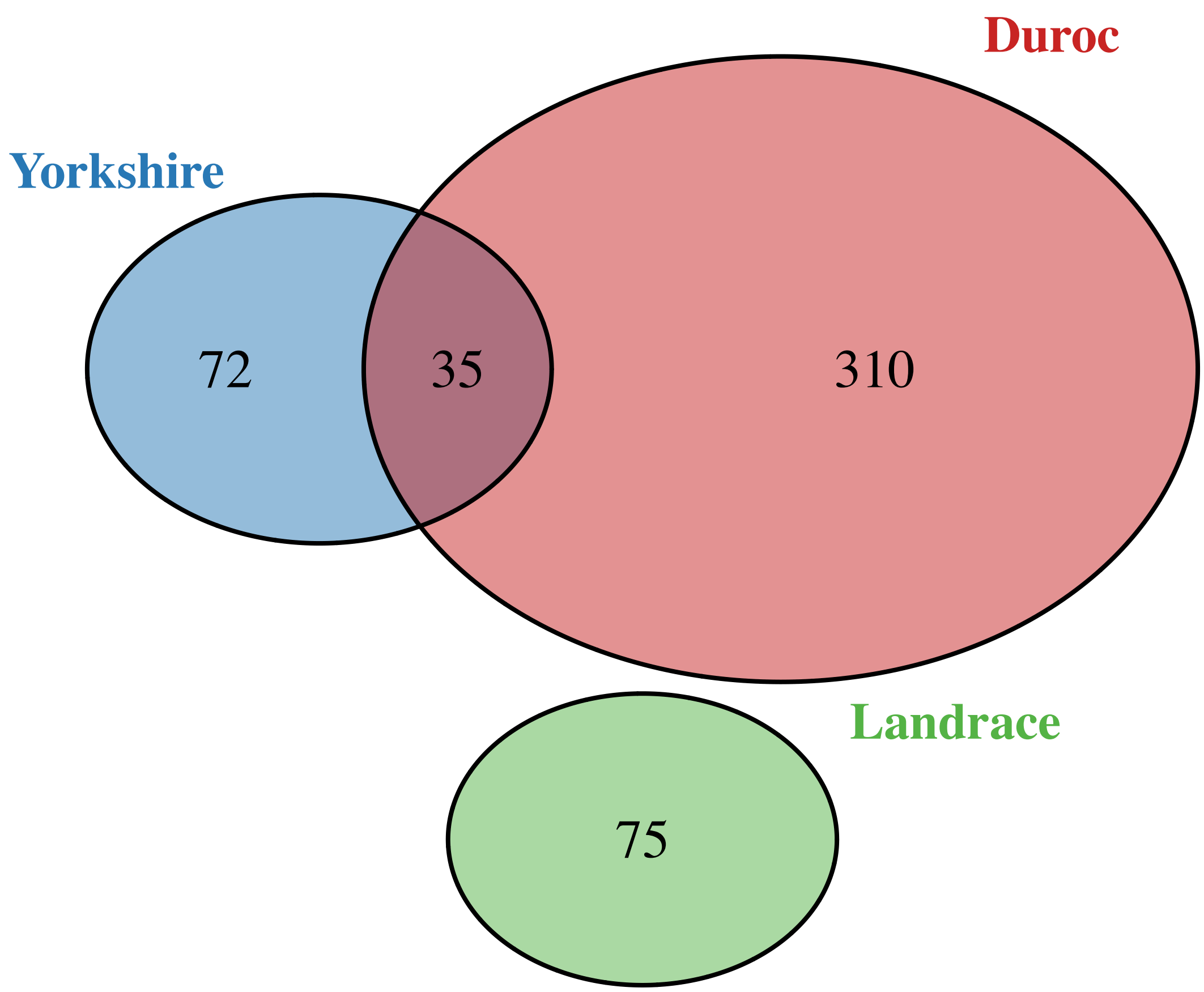

Supplementary_Figure_S3: Number of Shared Traits Information Across Duroc, Landrace and Yorkshire

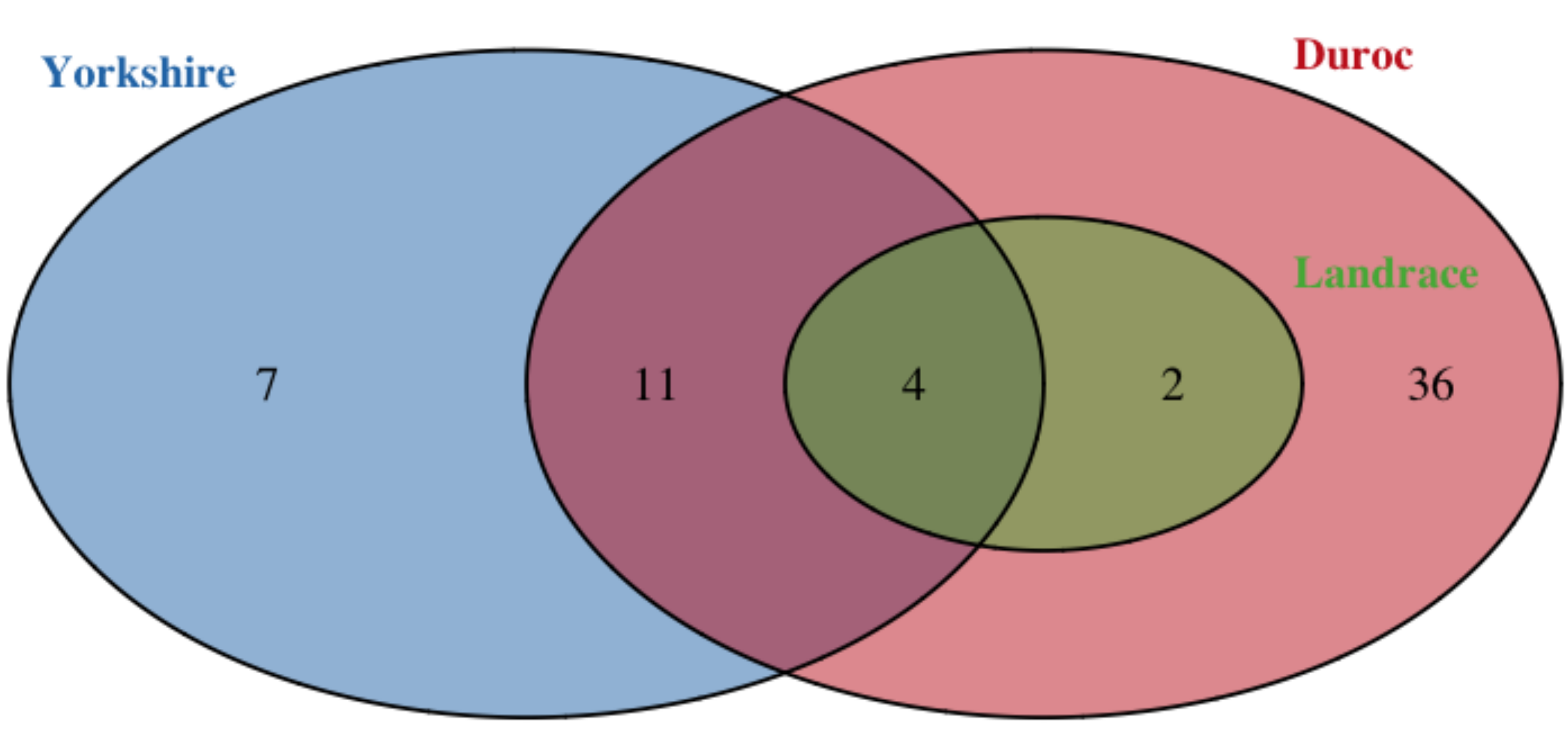

Supplementary_Figure_S4: Number of Shared Genes Across Duroc, Landrace and Yorkshire by ROHs analysis

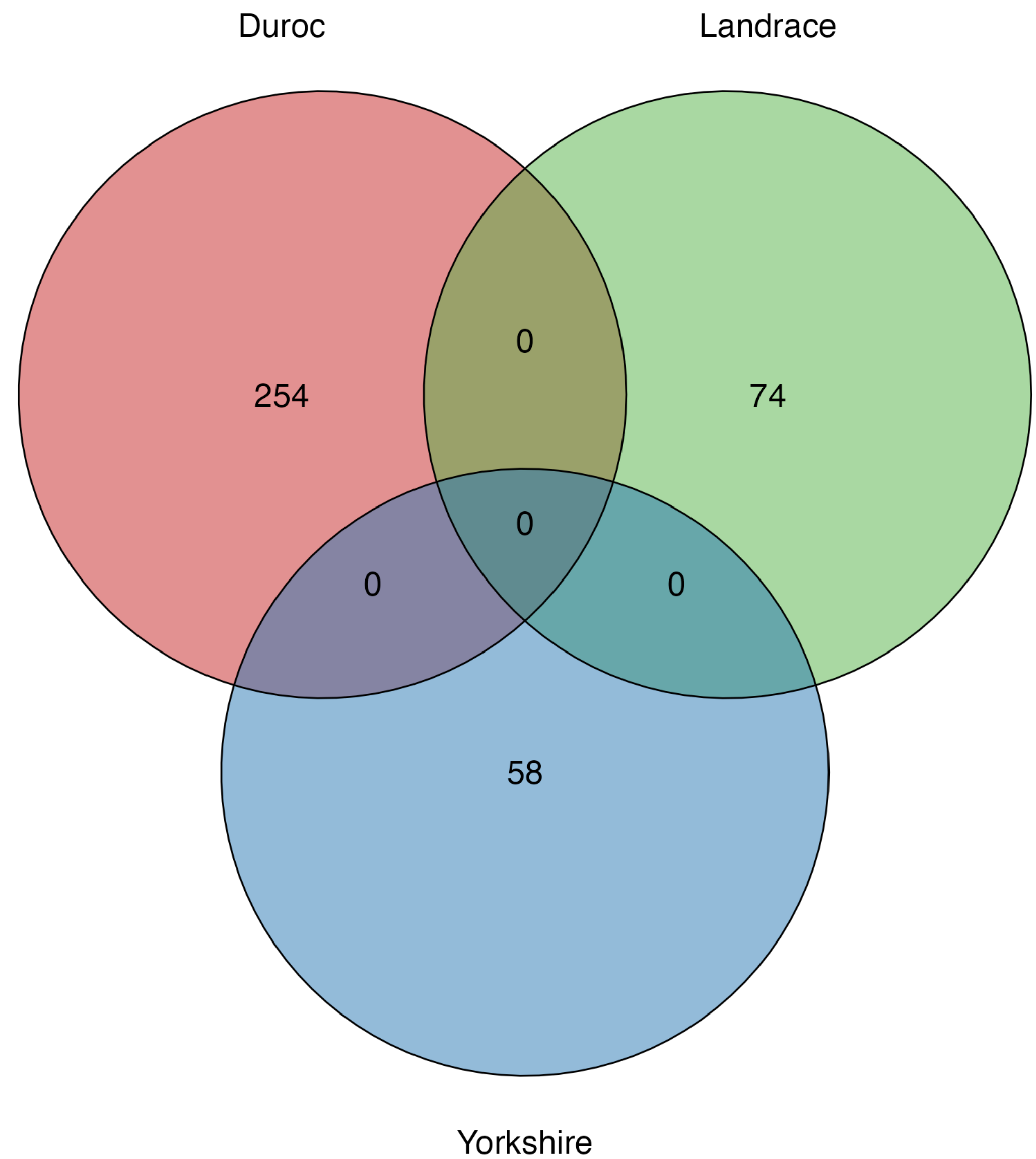

Supplementary_Figure_S5: Number of Shared Genes Across Duroc, Landrace and Yorkshire by GPSM analysis

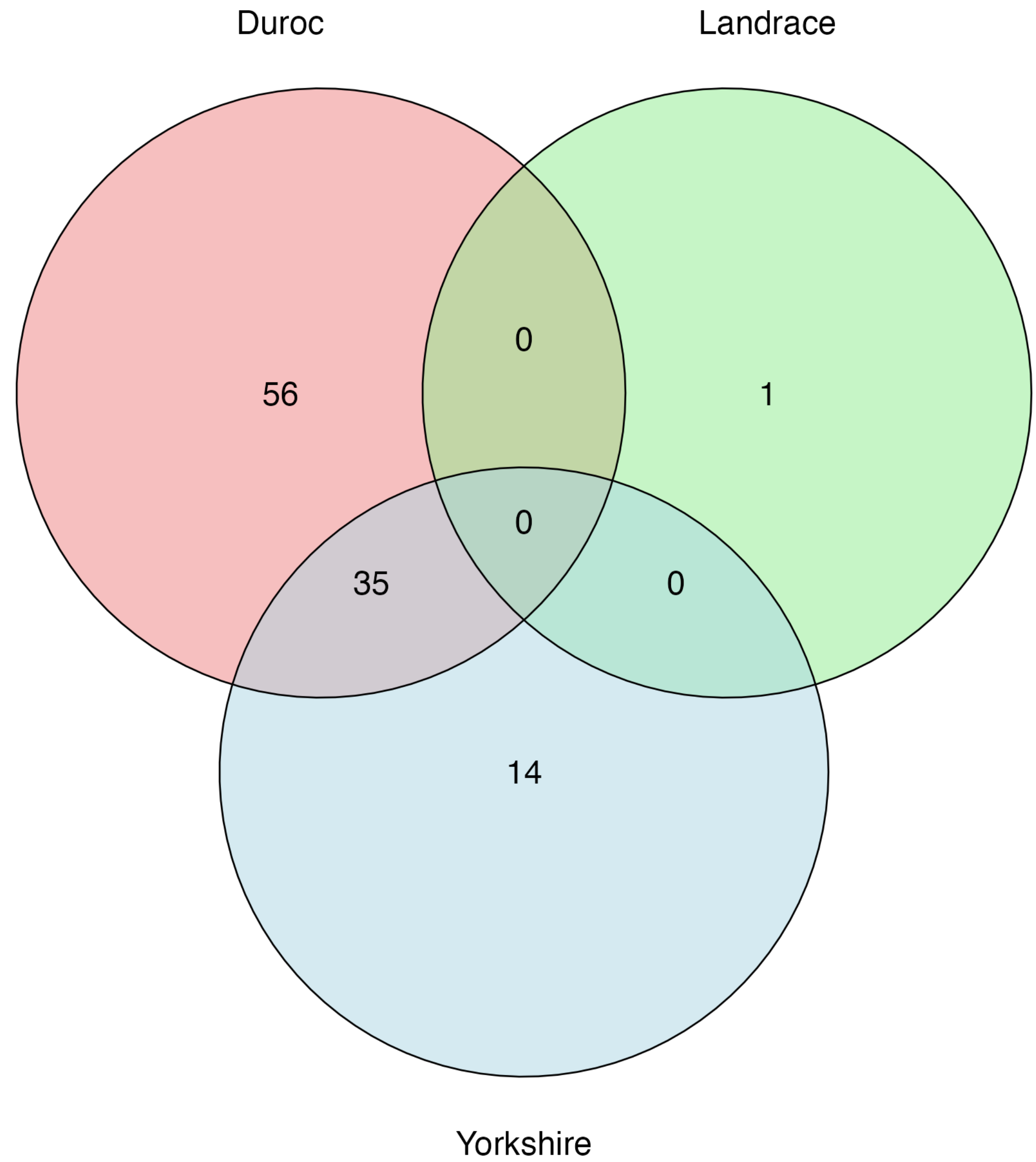

Supplementary_Figure_S6: Number of Shared Traits Information Across Duroc, Landrace and Yorkshire by ROHs analysis

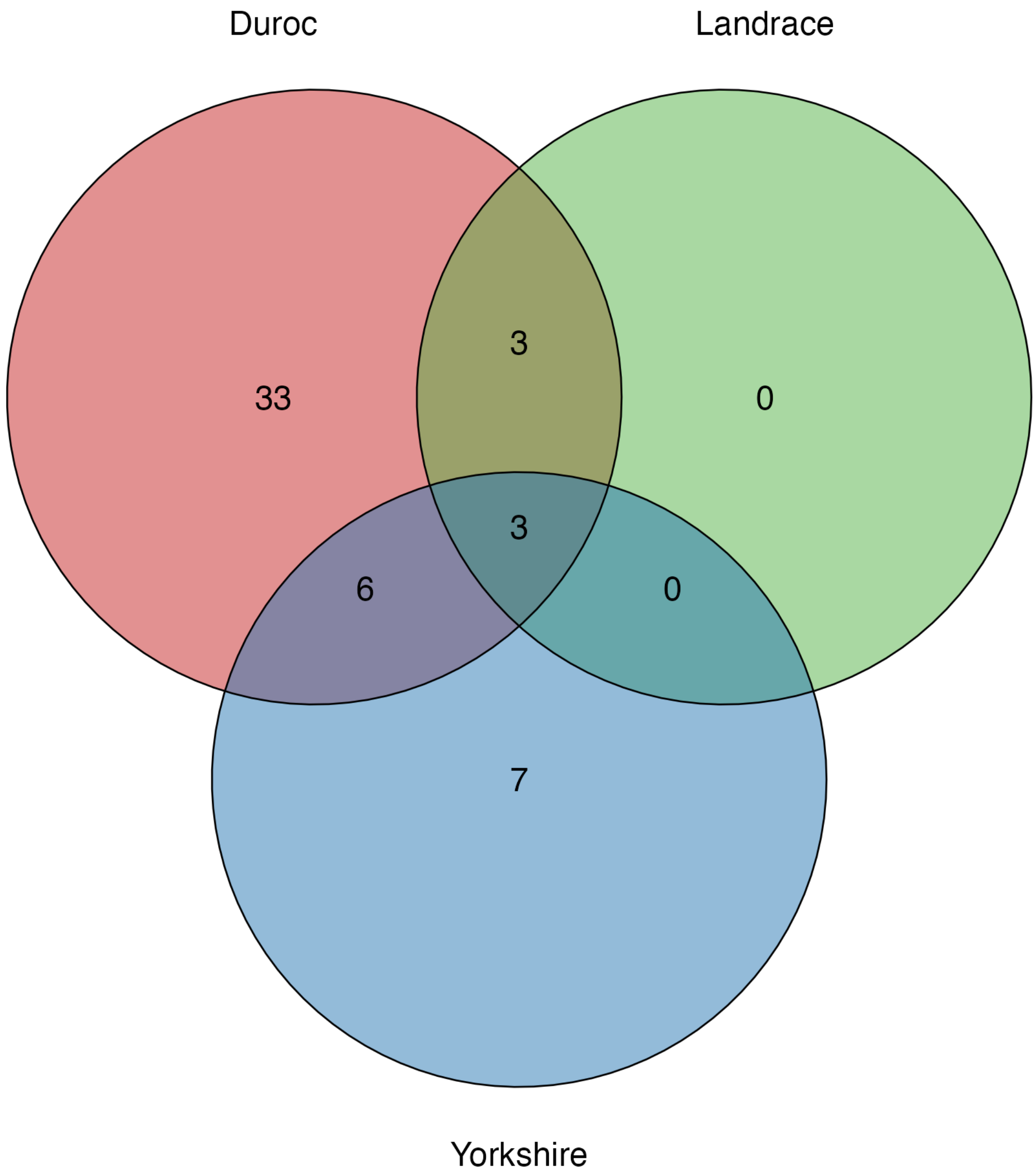

Supplementary_Figure_S7: Number of Shared Traits Information Across Duroc, Landrace and Yorkshire by GPSM analysis

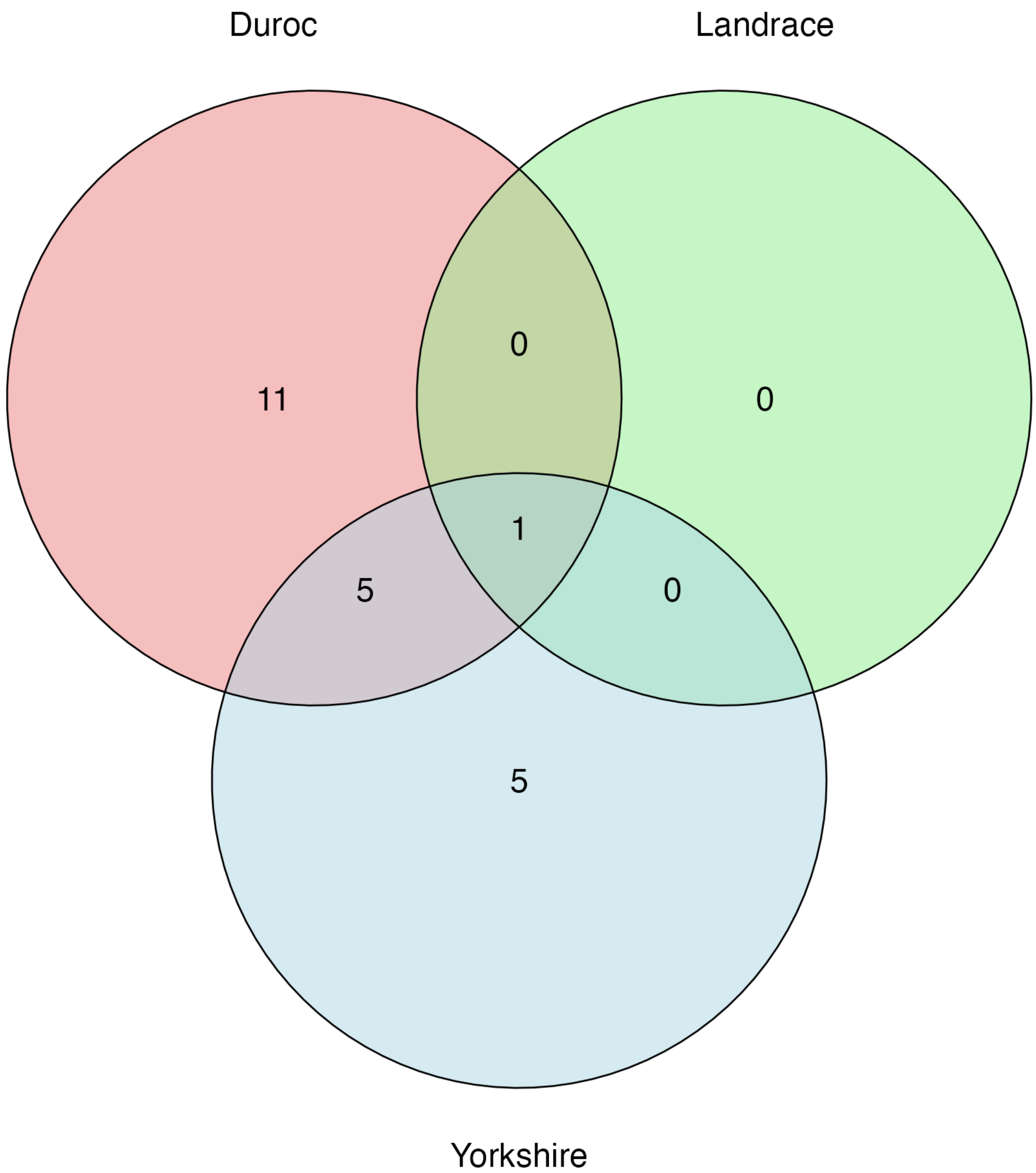

Supplementary Figure S8. Quantile–quantile (Q–Q) plots of GPSM P-values for three pig breeds: Duroc (A), Landrace (B), and Yorkshire (C)

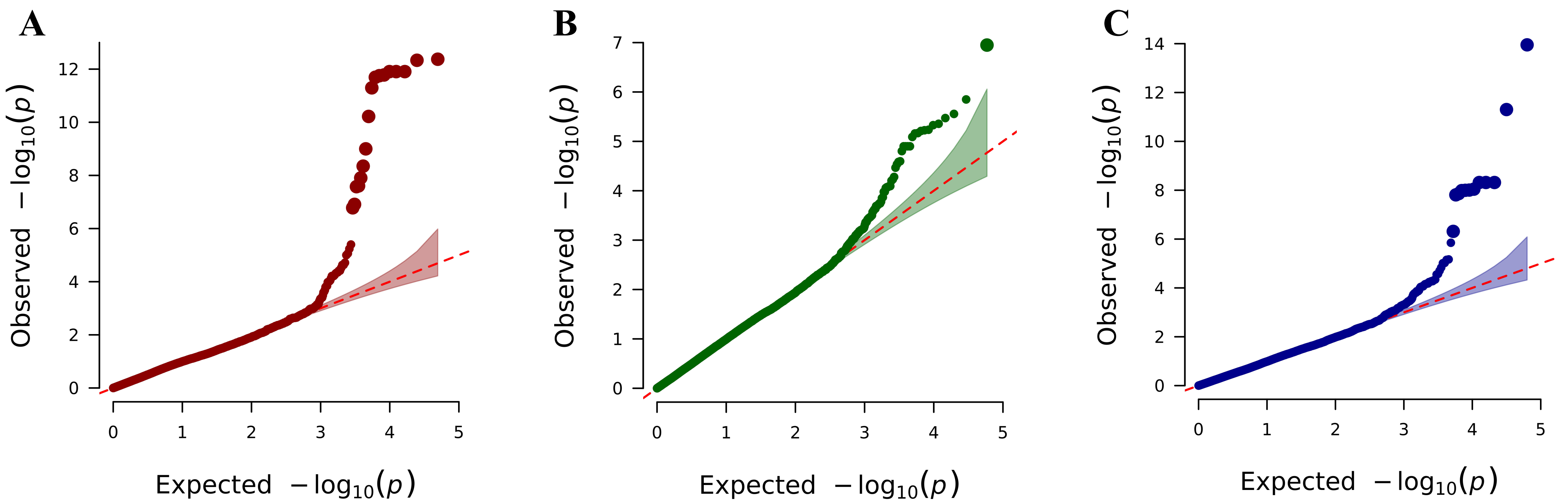

Supplement: Supplementary file 1 [file 13353_2026_1072_MOESM1_ESM.pdf]
